# What are the pros and cons of electronically monitoring inhaler use in asthma? A multistakeholder perspective

**DOI:** 10.1136/bmjresp-2016-000159

**Published:** 2016-11-29

**Authors:** Sam Howard, Alexandra Lang, Sarah Sharples, Dominick Shaw

**Affiliations:** 1 Human Factors Research Group, Innovation and Technology Research Centre, University of Nottingham, Nottingham, UK; 2 Division of Respiratory Medicine, School of Medicine, Nottingham City Hospital, University of Nottingham, Nottingham, UK

**Keywords:** Asthma, Inhaler devices

## Abstract

**Introduction:**

Electronic monitoring devices (EMDs) are the optimal method for collecting objective data on inhaler use in asthma. Recent research has investigated the attitudes of patients with asthma towards these devices. However, no research to date has formally considered the opinions of stakeholders and decision-makers in asthma care. These individuals have important clinical requirements that need to be taken into account if EMDs are to be successfully provisioned, making collecting their opinions on the key barriers facing these devices a valuable process.

**Methods:**

Three rounds of surveys in a Delphi format were used to assess the most important pros and cons of EMDs for asthma care in a sample of 31 stakeholders which included healthcare professionals and members of clinical commissioning groups.

**Results:**

The respondents identified 29 pros and 32 cons. Pros that were rated as most important included new visual evidence to aid clinical discussions with a patient and an increase in patient involvement and motivation. The cons that were rated as most important included a need for more clinical evidence of the effectiveness of EMDs, as well as better clarity over who has responsibilities in managing, interpreting and discussing data with a patient.

**Conclusions:**

The research provides a guide for EMD developers by highlighting where these devices may provide the most benefit as well as prioritising the key issues that need addressing if they are to be used effectively in everyday asthma care.

**Key messages**

- Electronic monitoring devices (EMDs) offer the most accurate solution for recording adherence to inhaled medication, yet little is known about the impact they could have on the healthcare system. This study examined the perceptions of healthcare providers and stakeholders in asthma care towards EMDs to examine what they believed their benefits could be, as well as their costs and barriers. The respondents felt that EMDs could promote better asthma control and better health for patients, and could also support decision-making and discussions with patients during clinical consultations. However, they also had concerns regarding cost, data governance and felt that more evidence was required for the effectiveness of these devices.

## Introduction

The 2014 National Review of Asthma Deaths found that children in the UK are still dying from avoidable asthma attacks, with widespread overprescribing of rescue medication a major issue.^1^ Moreover, adherence rates to inhaled steroids are below 75% in children and adolescents^2^ and asthma still costs the National Health Service (NHS) £1 billion a year.^3^

One option to improve compliance and potentially reduce reliever overuse is electronic monitoring devices (EMDs). A recent article referred to EMDs as the ‘21st century gold standard’ for measuring inhaler use.^4^ These devices are now considered the optimal method for accurately and reliably recording objective data on adherence for clinical and research practice.^2^ ^5^ Recent trials have extensively tested the validity and accuracy of exemplar EMDs over prolonged periods to fully demonstrate their efficacy for use in clinical research.^6^ ^7^ These devices have also been associated with improved adherence to inhaler therapy.^8^ ^9^ However, a recent review of currently available EMDs highlighted a lack of consideration for patient attitudes and stakeholder involvement as key issues facing these devices going forward.^10^

Initial evidence now suggests that adolescents feel positively towards the monitoring and reminding capabilities of EMDs for helping them to demonstrate their adherence and for ensuring they remember to take their inhaler on time, with their main concerns surrounding the bulkiness and unusual appearance of the devices.^11^ However, in order to improve asthma care at a population level, EMDs would also require interaction from a variety of healthcare professionals; all of whom have clinical requirements that need accounting for if the provision of a medical device is to ultimately be successful.^10^ ^12^ ^13^

The stakeholders who should have access to the data produced by EMDs have not been determined. However, if data indicating a patient's inhaler use was putting their health at risk were available to a healthcare professional, but was not acted on, they could be open to criticism for not intervening.^10^ This potentially major issue is highlighted by a recent clinical trial using EMDs where researchers reported ‘extreme overuse’ of β-agonists in 26% of their 152 participants, with these patients actuating 32 or more doses of reliever inhaler a day.^14^

To understand the potential benefits and issues that EMDs could create for the asthma care system, it is important to involve asthma stakeholders in the introduction of these devices, to help ensure they are ultimately safe and effective.

The aim of the present study was to use a three-round ‘Delphi’ Survey to engage with multiple asthma care stakeholders and decision-makers in order to understand what they perceive as the key pros and cons for the introduction of EMDs into everyday asthma management. It is envisaged that this information could be used to help inform and guide the future development and successful introduction of these devices into the NHS.

## Methods

### Defining stakeholders

The targeted study population—stakeholders—can be defined as ‘individuals, organisations or communities that have a direct interest in the process and outcomes of a project, research or policy endeavour’.^13^ For this research, stakeholders with an active interest in the future of asthma care were identified as being:

- *Healthcare providers*: General practitioners (GPs), nurses, consultants and pharmacists;
- *Payers and purchasers*: NHS clinical commissioning groups (CCGs);
- *Advisory boards*: British Thoracic Society (BTS) asthma specialist advisory group.

### Recruitment

As the targeted population was anticipated to be difficult to recruit from, several routes were used for contacting potential participants.

#### Paper survey

- Handed out to the respiratory teams at Queen’s Medical Centre and City Hospital in Nottingham, UK;
- Posted to members of local CCGs where they had listed their interests or specialties as ‘asthma’, ‘respiratory’ or ‘paediatrics’;
- Sent to members of the BTS asthma specialist advisory group;
- Handed out to delegates at the East Midlands Asthma Day (2015) at the University of Nottingham, UK.

#### Online survey

- Emailed to doctors and nurses in Leicestershire, UK;
- Emailed to members of the Respiratory Effectiveness Group (REG)—an investigator-led, not-for-profit research initiative (http://effectivenessevaluation.org);
- Shared on Twitter by the REG (@RespirEffect);
- Article and link to survey posted on the Respiratory Futures website (http://www.respiratoryfutures.org.uk).

### Study design

A ‘Delphi’ method was chosen as it is considered an effective research tool for collecting the judgments of experts in different physical locations.^15^ Through multiple rounds of surveys, this method also allows participants to view the anonymous opinions of others, then refine and adjust their own views dependent on their level of agreement.^16^ The three rounds of the Delphi Survey used in this study are explained further.

#### Delphi round one

Respondents were first given a short explanation of EMDs for asthma in case they were unaware of their purpose; this was carefully worded to avoid biases for or against the devices (see online supplementary appendix 1). They were then asked to provide at least six pros and six cons that they felt the introduction of EMDs could have for asthma care.

10.1136/bmjresp-2016-000159.supp1 supplementary appendix

#### Delphi round two

The responses from round one were collected and similar points were merged. For example, responses from participants such as ‘actual cost of device’, ‘increased inhaler costs’, ‘who funds the device?’ could all be grouped under the same point—‘cost of devices’. Participants were then provided with a randomised list of every unique point that was raised in round one, for pros and cons. Their task was then to rank each point for its level of importance, with ‘1’ being least important and ‘10’ being most important.

#### Delphi round three

The ratings from round two were then collated and analysed. Lists of the pros and cons ranked by their rated level of importance were then presented back to participants in round three and they were asked for final qualitative feedback on whether they agreed with the order, if they felt anything was missing, and if they felt that their views towards EMDs had changed over the process.

### Materials

Surveys were available in paper and online formats to allow for different methods of contacting potential participants. The online version of the survey was run using Qualtrics (https://www.qualtrics.com). The information included in both versions of the survey was the same.

## Results

### Study population

It was not possible to accurately estimate the number of potential participants compared with the number who responded because of the multiple different routes of recruitment that were used.

Participants recruited into the study all took part in round one of the Delphi, with dropout meaning a reduced number responded to round two and a further reduced number completed round three. Details of the sample at each stage are provided in table 1. Although there is no concrete recommendation for the sample size required for a Delphi Study, they are rarely conducted with <10 participants.^17^ The sample used here therefore falls within recommended guidelines.

**Table 1:** Sample size across the three rounds of the Delphi Survey with occupation demographics included

Delphi round	Total	Consultants	GPs	Nurses	Pharmacists	No occupation provided	CCG/advisory board members (from the existing sample)
Round one	31	8	6	9	1	7	5
Round two	18	8	3	6	1	0	4
Round three	10	3	1	6	0	0	1

CCG, clinical commissioning group; GP, general practitioner.

### Round one results

After collecting the responses for round one, the 154 pros and 159 cons of EMDs provided by the respondents were analysed to find occasions where different participants had raised the same point. Responses were then grouped and given names to accurately report every unique point raised by the participants.

A total of 29 pros and 32 cons were found from the data in survey one and are displayed in tables 2 and 3, respectively. The number of times each point was raised independently is shown in the tables and provides an indication of the factors that were brought up most frequently.

**Table 2:** The 29 pros participants gave for electronically monitoring inhaler use, with the number of times each point was raised

Pros	Sum
1. An accurate record of adherence for clinicians/nurses/general practitioners to use in auditing and review	24
2. Reminding the patient to use their inhaler	17
3. For identifying patterns of inhaler use, for example, days, times, school, holidays, etc	12
4. Aiding discussions between the clinician and patient, for example, visual evidence	11
5. Improve compliance	8
6. Reducing costs through less wasted medication and less time in hospital	8
7. Relating an accurate record of a patient’s inhaler use to their health outcomes and asthma control	7
8. Data for research	6
9. Patient can see their inhaler use from home and know if they are underusing/overusing	6
10. Patient has proof of their adherence to share with their clinician—increasing trust	6
11. Increase patient involvement and motivation for treating their condition	5
12. More informed decision-making for clinicians	5
13. Better asthma control and improved quality of life	4
14. Adding the ability to alert when the inhaler is about to run out would be beneficial	3
15. Adding the ability to monitor inhaler technique would be beneficial	3
16. Can be used to identify inhaler types that are less likely to be used—to ultimately find the most widely accepted and used inhaler types	3
17. Increasing patient independence, accountability and self-management for their asthma	3
18. Parents can check on their child's inhaler use	3
19. The patient's awareness of monitoring by their clinician may improve their compliance	3
20. ‘Cool’ technology may appeal to patients	2
21. Could reduce exacerbations	2
22. GPS would be beneficial in identifying triggers for a patient’s asthma, for example, pollen, pollution	2
23. Could be used with other monitoring techniques, for example, peak flow	1
24. Helpful for identifying dose dumping	1
25. Increasing patient confidence in their care	1
26. Long term—could be used to develop bio feedback	1
27. Promote competition	1
28. Useful data for emergency situations	1
29. Useful to monitor adherence of different groups of patients on different treatments	1

**Table 3:** The 32 cons participants gave for electronically monitoring inhaler use, with the number of times each point was raised

Cons	Sum
1. Cost of devices	32
2. Bulkiness and appearance may put patients off	14
3. Patient may not like being ‘watched’	12
4. Accuracy and reliability of the device, as well as potential technical issues	9
5. Concerns over the time and workload this would add to the consultation process	9
6. Concerns if this is only compatible with MDIs	7
7. Records actuation but not inhalation, technique, nor identifies if canister is empty or inhaler is shared	7
8. Whose responsibility is downloading, processing and interpreting the data and discussing with patients?	7
9. How is data stored and who has access?	6
10. An EMD may be required for more than one inhaler per patient	5
11. Evidence of the effectiveness of EMDs is required	4
12. Cleaning and maintenance of the device	3
13. Concerns about the role of pharma companies	3
14. Could interfere with inhalation technique or not be compatible with spacer	3
15. Data overload	3
16. Ease of use—another thing patients have to learn	3
17. Elderly patients may struggle with the technology or have a negative attitude towards it	3
18. May make no difference to already unengaged patients	3
19. May put patients off coming to clinic particularly if they have failed	3
20. Paternalistic approach	3
21. Added cost/time/workload of training clinicians and staff on how to use device, how to teach patients and how to interpret results	2
22. Are there better alternatives, for example, Tele-health or Medication Possession Ratio (MPR)?	2
23. Over-reliance on data—also need to determine reasons for non-adherence	2
24. Patient may forget to bring device with them to clinic	2
25. Patient resistance or refusal to use the device	2
26. Patients may find the reminders a nuisance	2
27. Bad for the environment—plastic and batteries	1
28. Could create potential conflicts between the patient and their clinician or parents	1
29. Many who get this device may do so as there are adherence concerns and therefore will show (inevitably) that adherence is poor	1
30. More benefits for researchers than patients, meaning patients may fail to see worth	1
31. Non-adopters lead to selection biases	1
32. This will not address intentional non-adherence	1

EMD, electronic monitoring device.

### Round two results

Participants in round two rated each point for its relative importance on a scale where 10 was most important and 1 was least important. To analyse these data, the number of 10, 9 and 8s were counted for each issue. The most important issues were then selected using the following criteria:

1. Total number of 10 and 9s;
2. If still equal between two or more issues—the total number of 10, 9 and 8s.

This calculation was done for the full sample of participants (N=18) as well as for the two largest subgroups—consultants (n=8) and nurses (n=6). The top five most important pros and cons for the full sample are shown in tables 4 and 5, with the data for consultants and nurses shown in table 6.

**Table 4:** The top five pros rated most important by the participants (10=most important, 1=least important)

Five most important pros (N=18)	10s, 9s	10s, 9s, 8s
1. Better asthma control and improved quality of life	9	13
2. Aiding discussions between the clinician and patient, for example, visual evidence	8	15
3. The patient's awareness of monitoring by their clinician may improve their compliance	7	14
4. Increase patient involvement and motivation for treating their condition	7	12
5. More informed decision-making for clinicians	7	11

**Table 5:** The top five cons rated most important by the participants (10=most important, 1=least important)

Five most important cons (N=18)	10s, 9s	10s, 9s, 8s
1. Evidence of the effectiveness of electronic monitoring devices is required	9	11
2. Records actuation but not inhalation, technique, nor identifies if the canister is empty or the inhaler is shared	6	11
3. Whose responsibility is downloading, processing and interpreting the data and discussing with patients?	6	10
4. Could interfere with inhalation technique or not be compatible with spacer	6	9
5. Patient may forget to bring device with them to clinic	6	7

**Table 6:** The top three most important pros and cons for the two occupation groups with the largest samples—consultants and nurses (10=most important, 1=least important)

	10s, 9s	10s, 9s, 8s
*Three most important pros*	*** ***	*** ***
Consultants (n=8)		
** **Aiding discussions between the clinician and the patient, for example, visual evidence	5	7
** **More informed decision-making for clinicians	5	6
** **The patient's awareness of monitoring by their clinician may improve their compliance	4	5
Nurses (n=6)		
** **Better asthma control and improved quality of life	4	5
** **Improve compliance	3	5
** **Relating an accurate record of a patient's inhaler use to their health outcomes and asthma control	3	3
*Three most important cons*		
Consultants (n=8)		
** **Evidence of the effectiveness of electronic monitoring devices is required	4	4
** **Patient may not like being ‘watched’	2	5
** **Could interfere with inhalation technique or not be compatible with spacer	2	4
Nurses (n=6)		
** **Whose responsibility is downloading, processing and interpreting the data and discussing with patients?	4	5
** **An electronic monitoring device may be required for more than one inhaler per patient	4	4
** **Accuracy and reliability of the device, as well as potential technical issues	4	4

As can be observed from the data, the points rated most important in round two are not the same points that were raised most frequently in round one. ‘Evidence of the effectiveness of EMDs is required’ was raised on only four occasions in the first round of the survey, yet was given a rating of 9 or 10 by half of the sample, making it the most important con facing EMDs, according to the results.

For consultants and nurses, there was a difference between the points they rated as most important. The pros rated important by the consultants appear to focus more on supporting the consultation process; through providing them with visual evidence to show patients—such as graphs on their adherence over a period of time, and by allowing them to make more informed decisions about a patient's care plan. In contrast, the pros rated highly by nurses focus more on the patient and their health, asthma outcomes and medication use. For cons, consultants rated ‘evidence of effectiveness of EMDs is required’ as most important, while nurses were more concerned with where responsibility would lie for downloading, interpreting and discussing the data with patients.

Interestingly, ‘records actuation but not inhalation, technique, nor identifies if canister is empty or inhaler is shared’ was rated as the second most important con overall (see table 5), yet was not rated in the top three cons for either consultants or nurses (see table 6). This was due to high ratings of 10 from two GPs, who were a smaller participant group.

### Round three results

In round three, the remaining 10 participants reviewed the ranked list of pros and cons formed from the ratings given in round two and provided their final comments and feedback.

Respondents provided new feedback on some of the pros and cons that had been formed in round one. For example, one consultant felt that ‘better asthma control and improved quality of life’ was not necessarily “…implicit in the use of smart inhalers [EMDs ]: it is what we hope will happen….” The same consultant felt that a pro that was missing from the list and should be considered important would be “Collecting data for making large scale decisions in terms of health services delivery and research.” Furthermore, the same consultant felt that a con that was currently missing but could be considered related to ‘cost of devices’ was ‘institutional procurement process’ and stated, “it is a complete pain to acquire things that aren't either drugs for a patient or equipment for the institute.”

One GP disagreed with EMDs being regarded as a ‘paternalistic approach’ and instead commented, “this is genuinely treating patients as an adult and empowering them.” The same participant felt that ‘data overload’ should be rated as more important, stating that this is “a genuine fear which needs to be addressed.” Elsewhere, a nurse felt that more should be made of the technology appealing to patients, “…particularly as we are often dealing with a younger adult age group.”

Many of the respondents spoke about how taking part in the research had changed their views towards EMDs. For example, one consultant said that their attitude had moved from “not at all interested [in using EMDs] to possibly interested for selected patients.” Whereas a nurse stated that their views were different because they now had a “…better understanding about how they work and the benefits to the patients.” However, another nurse who had previous experience with EMDs still had reservations about the devices, and believed they could often be prone to errors and faults, stating “these can be frustrating for both patients and clinicians, and may cause problems when reviewing results.”

### Overall review of the pros and cons associated with electronically monitoring inhaler use

Pros and cons were obtained which relate to the patient and their health, the job and workload of the clinician, advancing asthma research and the practicalities of EMDs more generally. An in-depth review of these factors is provided here, focusing on the factors where the most in-depth supporting quotes from participants were received.

#### Patient-related factors

Participants identified several pros and cons of EMDs that could directly affect the health and care of patients with asthma. The factors where the most in-depth comments were received are discussed here and a full list is provided in table 7.

**Table 7:** The pros and cons that are related to the patient

Patient-related pros	Patient-related cons
▸ Reminding the patient to use their inhaler	▸ Bulkiness and appearance may put patients off
▸ Improve compliance	▸ Patient may not like being ‘watched’
▸ Patient can see their inhaler use from home and know if they are underusing/overusing	▸ An electronic monitoring device may be required for more than one inhaler per patient
▸ Patient has proof of their adherence to share with their clinician—increasing trust	▸ Ease of use—another thing patients have to learn
▸ Increase patient involvement and motivation for treating their condition	▸ Elderly patients may struggle with the technology or have a negative attitude towards it
▸ Better asthma control and improved quality of life	▸ May make no difference to already unengaged patients
▸ Adding the ability to alert when the inhaler is about to run out would be beneficial	▸ May put patients off coming to clinic particularly if they have failed
▸ Increasing patient independence, accountability and self-management for their asthma	▸ Paternalistic approach
▸ Parents can check on their child's inhaler use	▸ Patient may forget to bring the device with them to clinic
▸ Patient's awareness of monitoring by their clinician may improve their compliance	▸ Patient resistance or refusal to use the device
▸ ‘Cool’ technology may appeal to patients	▸ Patient may find the reminders a nuisance
▸ Could reduce exacerbations	▸ Could create potential conflicts between the patient and their clinician or parents
▸ Increasing patient confidence in their care	▸ Many who get this device may do so as there are adherence concerns and this will show (inevitably) that adherence is poor
▸ Promote competition	▸ More benefits for researchers than patients, meaning patients may fail to see worth
	▸ This will not address intentional non-adherence

The respondents often spoke about how EMDs could have the potential to improve adherence, with some continuing to describe how this could lead to improved asthma control and quality of life. Comments included “compliance enhanced,” “Effective use of treatment,” “more likely to comply” and “Improved care, because of increased adherence.” Many of the respondents felt that the reminders an EMD could provide would have a direct effect on how often patients were using their inhalers. For example, one nurse stated, “The EMD would be useful in patients who forget to take their inhaler, and acts as a reminder, and get them into a habit of taking their inhaler regularly,” whereas another nurse commented, “Some children would love the fact that devices are being used to remind them….”

Participants also commented on the positive effect that EMDs could have for motivating patients and creating a sense of independence and accountability for their condition. A nurse commented that “ the patients can individualise the EMD by selecting their own tune and time of the alarm, which by involving the patient in choice may increase adherence to medication” while a pharmacists and CCG member stated “Uploadable results may improve patient involvement and interest in their treatment.” Some comments were also received from respondents who felt that the technology could be an attractive feature, such as “The device may appeal to patients who like the new technology and gadget feel.”

Participants also felt that the data recorded by EMDs could foster a trusting relationship between the patient and their clinician. For example, one respiratory consultant stated, “patients know that clinicians ‘believe ’ them re concordance” while a GP similarly said that it “may encourage asthma review as have ‘done well ’ and will receive praise.” Some respondents also felt that a patient simply being aware of their inhaler use being monitored could have a positive impact, with a respiratory consultant commenting, “Positive observation effect: people won't know when they are being watched so are more likely to behave.”

While participants recognised the benefit EMDs could have for patients with asthma, they equally had concerns for the potential barriers that may need to be addressed or overcome for benefits to come to fruition.

First, many comments pertaining to the bulkiness and size of the device were received. One GP and CCG member stated that the “EMD may make the whole inhaler bulky—not user friendly and may discourage patients to clip it onto their inhaler,” while a respiratory consultant with first-hand experience of EMDs warned, “they were not popular with patients, many liked the idea but found the reality of the device too bulky and cumbersome.” Others additionally felt that the usability of the device could be a potential issue, with a nurse and CCG member commenting, “this may be another device the patient has to get to grips with along with the various types of inhaler they may be using.” This could be a particular problem in older patients; a GP and CCG member asked, “Are they accessible to the older generation who may not be quite so ok with using technology?”

As opposed to the potential benefits for some patients in encouraging good adherence, respondents felt there was potential for EMDs to have a negative effect on a patient's attitude. A GP commented, “Intrusion may not be well received—‘Big Brother’” and a nurse stated, “people are highly resistant to being watched.” Some felt this could even effect whether patients attend clinic visits, with a respiratory consultant stating, “Patients may feel their clinician is checking up on them and may be put off coming for review.”

Comments were also received stating that this was a “paternalistic approach to medicine” and a “restriction of a patient's autonomy.” However, this was not a universal opinion.

#### Clinician-related factors

With the data collected by an EMD having wide-ranging uses and implications for a clinician and their care of patients; the participants identified many different pros and cons related to healthcare professionals. The factors where the most in-depth comments were received are discussed here and a full list is provided in table 8.

**Table 8:** The pros and cons that are related to the clinician

Clinician-related pros	Clinician-related cons
▸ An accurate record of adherence for clinicians/nurses/general practitioners to use in auditing and review	▸ Concerns over the time and workload this would add to the consultation process
▸ For identifying patterns of inhaler use, for example, days, times, school, holidays, etc	▸ Whose responsibility is downloading, processing and interpreting the data and discussing with patients?
▸ Aiding discussions between the clinician and the patient, for example, visual evidence	▸ Data overload
▸ Relating an accurate record of a patient's inhaler use to their health outcomes and asthma control	▸ Added cost/time/workload of training clinicians and staff on how to use device, how to teach patients and how to interpret results
▸ More informed decision-making for clinicians	▸ Over-reliance on data—also need to determine reasons for non-adherence
▸ GPS would be beneficial in identifying triggers for a patient's asthma, for example, pollen, pollution	
▸ Helpful for identifying dose dumping	
▸ Useful data for emergency situations	

Many of the participants spoke about the devices providing an accurate record of adherence that could be used by a clinician in their auditing of a patient. For example, one respiratory consultant said that EMDs would create “a tool to monitor actual adherence achieved against the goals previously agreed.” Some respondents specifically mentioned how this would be a more accurate estimate of inhaler use than asking the patient directly, with one CCG member saying it would “improve assessment of compliance—rather than rely on a patient telling their clinician.”

Participants then spoke of how this accurate record of adherence would be useful for identifying patterns of use, with one nurse and CCG member stating, “Being able to demonstrate patterns of use… is particularly important for patients who forget to use their inhalers or are unsure of the frequency of use for ‘as required’ inhalers.” Other participants then additionally spoke about how a true record of inhaler use could be related to health outcomes and asthma control, “the data could be used to establish if their asthma control is poor due to reduced adherence to medication or their medication requires escalation to improve control.”

Furthermore, participants commented on how this detailed picture of a patient's medication management would ultimately lead to more informed decision-making, with one respiratory consultant stating it would provide “objective data for clinicians to make decisions, not report” and another consultant adding, “Changes in medication…could be based on accurate reports of current medication regimen administered.” There was also a shared feeling that the data could be used to aid discussions between a clinician and patient. One nurse provided the insightful comment that “We do practice open conversations, but seeing things written down or pictorially presented may be more effective in devising an action plan together.”

Much the same as with factors related to the patient, the participants had concerns about the potential barriers EMDs may have to overcome to provide true benefit for clinicians.

First, there were concerns expressed by participants about the time and added workload EMDs could create. One nurse said, “The time to download and examine the data collected from the EMDs in clinic may be limited,” while a GP and CCG member stated, “Doctors don’t have enough time to follow up adherence.”

Questions were also asked about who should actually take responsibility for downloading, processing and interpreting the data and then discussing it with the patient. One participant stated, “some clinicians may not appreciate that the time taken to discuss results together is actually saving time in the future. Who is the best professional to discuss data with patient/family? GP, nurse, specialist doctor or other?” Related to this was a comment about the cost of training, “clinicians will require training on the use and interpreting of the results.”

#### Research-related factors

With a proportion of the recruited sample having strong ties to academic research, points were raised in relation to using EMDs for asthma research going forward, as well as further research required now. The factors where the most in-depth comments were received are discussed here and a full list is provided in table 9.

**Table 9:** The pros and cons that are related to research

Research-related pros	Research-related cons
▸ Data for research	▸ Evidence of the effectiveness of electronic monitoring devices is required
▸ Can be used to identify inhaler types that are less likely to be used—to ultimately find the most widely accepted and used inhaler types	▸ Are there better alternatives, for example, Tele-health or Medical Possession Ratio (MPR)?
▸ Long-term could be used to develop bio-feedback	▸ Non-adopters lead to selection biases
▸ Useful to monitor adherence of different groups of people on different treatments	

One respiratory consultant felt that EMDs could be useful “to monitor concordance of different groups of patients on different treatment regimens” to examine how adherence varies from medication to medication. Along a similar line, a GP commented that EMDs could be used to “identify devices which are less likely to be used and weed them out, and which ones are easier and more likely to be used.”

Another GP spoke of the potential for location monitoring, stating it could be useful “to identify triggers…but may not be accurate if trigger is mobile.” Elsewhere, a nurse recognised the benefit EMDs could have for research more generally, “to validate study results by showing compliance with a particular treatment.”

Although research exists that demonstrates the accuracy of EMDs, some participants felt that more evidence is needed. One respiratory consultant asked, “Has the data they collect been validated? Personal use with an older generation of these devices [on a research study] has been poor. Ranged from recording puffs for patients who had never used them to recording nothing at all when we knew they were definitely used.” Another participant asked, “does the EMD have a sustainable effect as a reminder?”

#### Practical factors

Many of the points raised by the participants were applicable to the healthcare industry in general and focused on the practicalities of the devices. The factors where the most in-depth comments were received are discussed here and a full list is provided in table 10.

**Table 10:** The pros and cons that are related to practical factors

Practical-related pros	Practical-related cons
▸ Reducing costs through less wasted medication and less time in hospital	▸ Cost of devices
▸ Could be used with other monitoring techniques, for example, peak flow	▸ Accuracy and reliability of the device, as well as potential technical issues
	▸ Concerns if this is only compatible with MDIs
	▸ Records actuation but not inhalation, technique, nor identifies if canister is empty or inhaler is shared
	▸ How is data stored and who has access?
	▸ Cleaning and maintenance of the device
	▸ Concerns about the role of pharma companies
	▸ Could interfere with inhalation technique or not be compatible with spacer
	▸ Bad for the environment—plastic and batteries

Some participants recognised that better adherence and asthma control could lead to a reduction in hospital admissions, wasted medication and healthcare costs. One nurse and CCG member stated, “Ensuring better adherence to inhaler treatment will have a positive effect on wider health economics, eg, more patients being well managed.”

While the potential positive financial effects of EMDs were recognised by some participants, many had concerns about the initial cost of the devices. One nurse asked, “How much do they cost? Is this cost incurred by the department, GP practise or patient?” while a GP stated, “The cost of each EMD may be expensive and may be limited to the more severe asthmatics having them.” The points were typically questioning of the cost rather than dismissive of it, highlighting a need for better clarity on initial costings versus potential savings.

There were also some concerns regarding the reliability of the devices with a nurse asking, “How robust are they? They need to be up to being thrown around and chucked in bags or drawers and left in wet steamy bathrooms!” Participants also raised issue with the fact the devices typically only record actuation and do not record inhalation, technique nor detect if the inhaler is shared. One participant stated, “I would want assurance that the device was not open to abuse, eg, removable in order to conceal overuse or inhaler sharing, or pressing device and not inhaling.”

## Discussion

### Implications for EMDs and asthma care

By examining the pros and cons of electronically monitoring inhaler use from the perspective of stakeholders, this research provides a knowledge base for EMD developers. The data gathered should be used as a guide to help inform future directions for improving the design of the devices and for providing greater clarity to stakeholders for how they could realistically be integrated into everyday asthma care. For example, participants here expressed concerns about the time these devices could add to the consultation process, as well as the training that would be required to use them. EMD developers should work alongside healthcare professionals to ensure devices and accompanying software are simple and intuitive to reduce their impact on consultation time.

### Limitations and future research

The sample used here was sufficient to gather a comprehensive list of the main pros and cons associated with EMDs, but was limited in the number of participants who gave ratings for each point's importance. Furthermore, the sample contained more consultants and GPs than nurses, meaning a balance of different viewpoints may not have been fully achieved. Future research should look to distribute the list of pros and cons to a larger sample, such as a respiratory organisation, to gather a larger data set for the issues stakeholders rate as most crucial to address.

Future research should also address data governance. While participants here did question whose responsibility it would be to download, interpret and discuss the data with patients, this should be examined further. Working with stakeholders to establish how data could realistically be handled and managed within the NHS will help to reduced fears of ‘data overload’ and ensure that the data can be used appropriately and effectively to ultimately benefit patient care.

## Conclusion

Through collecting the views of stakeholders with a direct interest in the future of asthma care, a comprehensive list of the pros and cons associated with introducing EMDs into the NHS has been obtained. The stakeholders raised points relating to the care and well-being of patients, the work of a clinician, research and more practical factors such as device reliability and cost. Factors that particularly need addressing by EMD developers include assurances over the cost of the devices and data governance. The findings provide a guide for where EMDs could add most benefit and additionally identify the key issues and challenges that need to be overcome for their introduction to be successful.

## References

[1] NRAD. Why asthma still kills: The National Review of Asthma Deaths (NRAD). London, UK, 2014.

[2] Morton RW, Everard ML, Elphick HE Adherence in childhood asthma: the elephant in the room. Arch Dis Child 2014;99:949–53. doi:10.1136/archdischild-2014-306243 24876303 10.1136/archdischild-2014-306243

[3] Asthma UK Asthma Facts and Statistics. Asthma.org.uk 2016.

[4] Bush A, Fleming L Is asthma overdiagnosed? Arch Dis Child 2016;101:688–9. doi:10.1136/archdischild-2015-309053 27049005 10.1136/archdischild-2015-309053

[5] Klok T, Kaptein AA, Brand PL Non-adherence in children with asthma reviewed: the need for improvement of asthma care and medical education. Pediatr Allergy Immunol 2015;26:197–205. doi:10.1111/pai.12362 25704083 10.1111/pai.12362

[6] Patel M, Pilcher J, Chan A Six-month in vitro validation of a metered-dose inhaler electronic monitoring device: implications for asthma clinical trial use. J Allergy Clin Immunol Pract 2012;130:1420–2. 10.1016/j.jaci.2012.06.037 22920492

[7] Pilcher J, Shirtcliffe P, Patel M Three-month validation of a turbuhaler electronic monitoring device: implications for asthma clinical trial use. BMJ Open Respir Res 2015;2:e000097 doi:10.1136/bmjresp-2015-000097 10.1136/bmjresp-2015-000097 PMC4653861 26629345

[8] Foster JM, Usherwood T, Smith L Inhaler reminders improve adherence with controller treatment in primary care patients with asthma. J Allergy Clin Immunol 2014;134:1260–8. doi:10.1016/j.jaci.2014.05.041 25062783 10.1016/j.jaci.2014.05.041

[9] Chan AH, Stewart AW, Harrison J The effect of an electronic monitoring device with audiovisual reminder function on adherence to inhaled corticosteroids and school attendance in children with asthma: a randomised controlled trial. Lancet Respir Med. 2015;3:210–19. doi:10.1016/S2213-2600(15)00008-9 25617215 10.1016/S2213-2600(15)00008-9

[10] Howard S, Lang A, Patel M Electronic monitoring of adherence to inhaled medication in asthma. Curr Respir Med Rev 2014;10:50–63.

[11] Howard S, Lang A, Youle C Exploring the attitudes of adolescents with asthma towards monitoring and sharing of data on their inhaler use. Eur Respir J 2015.

[12] Grocott P, Weir H, Ram MB A model of user engagement in medical device development. Int J Health Care Qual Assur 2007;20:484–93. 18030966 10.1108/09526860710819422

[13] Deverka PA, Lavallee DC, Desai PJ Stakeholder participation in comparative effectiveness research: defining a framework for effective engagement. J Comp Eff Res 2012;1:181–94. doi:10.2217/cer.12.7 22707880 10.2217/cer.12.7 PMC3371639

[14] Patel M, Pilcher J, Pritchard A Efficacy and safety of maintenance and reliever combination budesonide–formoterol inhaler in patients with asthma at risk of severe exacerbations: a randomised controlled trial. Lancet Respir 2013;1:32–42. 10.1016/S2213-2600(13)70007-9 24321802

[15] Czinkota M, Ronkainen I International business and trade in the next decade: report from a Delphi study. J Int Bus Stud 1997;17:827–44.

[16] Rowe G, Wright G The Delphi technique as a forecasting tool: issues and analysis. Int J Forecast 1999;15:353–75.

[17] Akins RB, Tolson H, Cole BR Stability of response characteristics of a Delphi panel: application of bootstrap data expansion. BMC Med Res Methodol 2005;5:37 doi:10.1186/1471-2288-5-37 16321161 10.1186/1471-2288-5-37 PMC1318466

