# Supplementary material for: What are the pros and cons of electronically monitoring inhaler use in asthma? A multistakeholder perspective
Source: BMJ Open Respir Res. 2016 Nov 29;3(1):e000159. doi: 10.1136/bmjresp-2016-000159 (PMC5133420; doi:10.1136/bmjresp-2016-000159)
Supplement: supplementary appendix [file bmjresp-2016-000159supp_appendix.pdf]

Appendix 1 – Information on EMDs presented to participants

EMD Information.

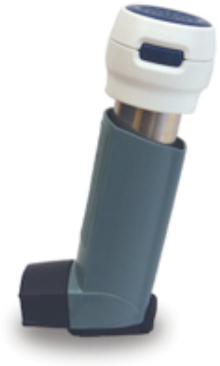

Propeller Sensor

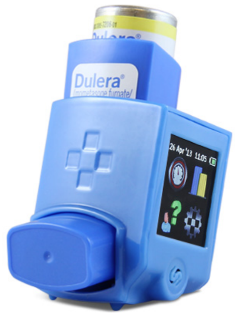

SmartTouch AV

The devices above are both examples of EMDs that can be used in conjunction with a patient's normal inhaler; by either clipping on top of the canister or clipping around the plastic actuator.

The key function of all EMDs is to precisely record the exact date and time whenever the inhaler is used.

This data can then be uploaded to a dedicated website where the patient or anyone with granted access can view detailed information on exactly when that individual has been using their inhaler.

Some EMDs possess additional features, such as audible tones to remind a patient when their next dose is due and GPS location tracking to identify the exact location of each actuated dose, to offer indication of particular asthma triggers.

Specific information on the benefits and costs of EMDs has been avoided in this description as much as possible in order to reduce the likelihood of influencing your responses.

Below are some suggestions for things you may wish to think about when deciding on your main benefits and costs/barriers in the following survey:

- Who could use the data collected by EMDs?
- What could the data collected by EMDs be used for?
- How would this wealth of data affect asthma treatment in its current form?
- Could EMDs have a negative impact on asthma care? If so, how?
